# Female Deer Movements Relative to Firearms Hunting in Northern Georgia, USA

**DOI:** 10.3390/ani14081212

**Published:** 2024-04-18

**Authors:** Jacalyn P. Rosenberger, Adam C. Edge, Charlie H. Killmaster, Kristina L. Johannsen, David A. Osborn, Nathan P. Nibbelink, Karl V. Miller, Gino J. D’Angelo

**Affiliations:** 1 Daniel B. Warnell School of Forestry and Natural Resources, University of Georgia, Athens, GA 30602, USAgdangelo@uga.edu (G.J.D.); 2 Game Management Section, Wildlife Resources Division, Georgia Department of Natural Resources, Social Circle, GA 30025, USA

**Keywords:** Appalachian, deer behavior, hunter pressure, hunting, management, movement ecology, national forest, public land, spatial ecology, white-tailed deer

## Abstract

**Simple Summary:**

Hunting can have direct effects (i.e., mortality) and indirect effects (i.e., behavior changes, such as shifting away from foraging areas to circumvent risk from hunters) on white-tailed deer (*Odocoileus virginianus*). Deer populations within the Chattahoochee National Forest of northern Georgia, USA, have declined significantly since the 1980s. Since deer population sustainability is a concern, understanding the potential negative effects of hunting on female deer is important. During the 2018–2019 and 2019–2020 hunting seasons, we evaluated the indirect effects of 7 firearms hunts for male deer on 20 non-target female deer. We used GPS locations recorded every 30 min during pre-hunt, hunt, and post-hunt periods to calculate and compare movement rates during the day and night, as well as the size and landscape characteristics of the space the deer utilized. Our results suggest that the low level of hunting pressure in our study area led to no biologically significant changes in female deer movements. To the extent of the findings presented in this paper, adjustments to the management of hunting in our study area do not appear to be necessary to minimize hunting-related disturbances for female deer. However, managers should continue to consider female deer behavior when evaluating future changes to hunting regulations.

**Abstract:**

Perceived risk associated with hunters can cause white-tailed deer (*Odocoileus virginianus*) to shift their activity away from key foraging areas or alter normal movements, which are important considerations in managing hunting and its effects on a population. We studied the effects of seven firearms hunts on the movements of 20 female deer in two Wildlife Management Areas within the Chattahoochee National Forest of northern Georgia, USA, during the 2018–2019 and 2019–2020 hunting seasons. Deer populations and the number of hunters in our study area have declined significantly since the 1980s. In response, hunting regulations for the 2019–2020 hunting season eliminated opportunities for harvesting female deer. To evaluate the indirect effects of antlered deer hunting on non-target female deer, we calculated 90% utilization distributions (UDs), 50% UDs, and step lengths for pre-hunt, hunt, and post-hunt periods using the dynamic Brownian bridge movement model. Data included 30 min GPS locations for 44 deer-hunt combinations. Pre-hunt 50% UDs ($\overset{-}{x}\;$= 7.0 ha, SE = 0.4 ha) were slightly greater than both hunt ($\overset{-}{x}\;$= 6.0 ha, SE = 0.3 ha) and post-hunt ($\overset{-}{x}$ = 6.0 ha, SE = 0.2 ha) 50% UDs (*F* = 3.84, *p* = 0.03). We did not detect differences in step length, nor did we detect differences in size or composition of 90% UDs, among the periods. Overall, our results suggest that the low level of hunting pressure in our study area and lack of exposure to hunters led to no biologically significant changes in female deer movements. To the extent of the findings presented in this paper, adjustments to the management of hunting in our study area do not appear to be necessary to minimize hunting-related disturbances for female deer. However, managers should continue to consider female deer behavior when evaluating future changes to hunting regulations.

## 1. Introduction

Hunting is effective for manipulating game populations in line with management objectives [1], as well as informing population models and indicating population trends [2]. In addition to the direct impact of hunting on populations, hunting can also impact game indirectly by altering behavior [3,4,5]. In a low-density white-tailed deer (*Odocoileus virginianus*) population where antlerless harvest is limited or prohibited, hunting pressure associated with antlered deer or other game species may alter non-target female deer movements that hinder normal activities, such as procurement of food resources or breeding, as has been reported for elk (*Cervus canadensis*; [6]) and mule deer (*Odocoileus hemionus*; [7]). These alterations may have negative population effects; therefore, where deer population sustainability is a concern, understanding the effects of hunting on female activity can assist managers in minimizing disturbance on female deer.

Perceived risk associated with hunters on the landscape can cause deer to shift their activity away from key foraging areas to those associated with lower risk [6,8,9]. Male European red deer (*Cervus elaphus*) in central Norway utilized different habitats when hunters were present on the landscape, trading off the best foraging opportunities for increased survival [9]. Female elk in Oregon, USA, shifted their habitat use away from areas of optimal forage during hunts, which had significant nutritional costs, especially for lactating females [6]. Infrequent periods of high risk can also cause deer to devote less energy to activities related to reproduction [10] or alter normal activities related to breeding [11,12]. Stress associated with perceived risk can induce physiological responses that suppress reproduction [13,14]. Hunting pressure in a low-density deer population that causes female deer to decrease movements could disrupt their normal mate-searching approach (i.e., increased movements) during the breeding season [15,16]. Male white-tailed deer in southcentral Oklahoma, USA, decreased movements due to hunting pressure when increased movements were expected due to breeding activity, suggesting there was a trade-off between risk avoidance and reproduction [11,12].

The goal of our research was to determine whether management actions to alter hunter distribution were necessary to minimize disturbance of female white-tailed deer on public land in the Southern Appalachian Mountains of northern Georgia, USA. We studied the movements and space use of female deer before, during, and after multiple firearms hunts over a 2-year period. Our research offered a unique management situation where both the deer population and the number of hunters experienced a history of decline in Wildlife Management Areas (WMAs) within the Chattahoochee National Forest. Deer density estimates dropped from 7.7 deer/km^2^ on Coopers Creek WMA and 8.9 deer/km^2^ on Blue Ridge WMA in 1992 [17] to 1.9–3.9 deer/km^2^ on both WMAs in 2018 [18]. Concurrently, the number of hunters on the WMAs decreased by 74%, and harvest success rates (i.e., antlered deer harvested/hunter/day) declined by 35% [19]. Thus, deer populations reached a critical level on the WMAs, leading to extremely limited opportunities for antlerless harvest during the first season of our study (2018–2019) and the prohibition of antlerless harvest during the second season (2019–2020). However, multiple hunts were still conducted on each WMA for antlered deer, in which black bears (*Ursus americanus*) and wild pigs (*Sus scrofa*) were also legal for harvest. Therefore, even though female deer were not available for harvest, understanding the effects of hunting on their movement patterns would help managers determine whether steps to manipulate hunting pressure would be warranted. Due to the drop in hunter participation, we predicted that hunting pressure would likely be insufficient to cause changes in deer behavior. However, due to the fact that hunters typically focus on areas where deer are more active, it is possible that despite the lower number of hunters, deer still experienced significant hunting pressure.

## 2. Materials and Methods

### 2.1. Study Site

**Figure 1:**
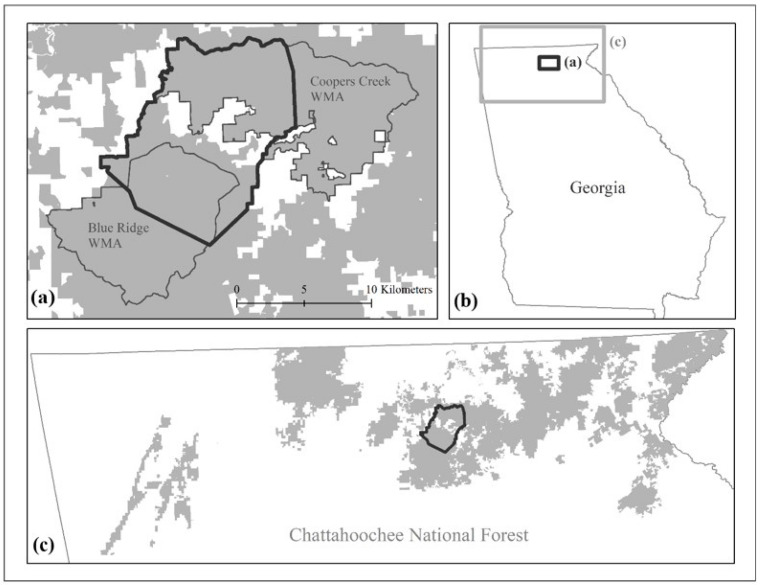
The 135 km^2^ study area (dark boundary in (**a**,**c**)) for tracking movements of 20 GPS-collared female white-tailed deer relative to firearms hunts during the 2018–2019 and 2019–2020 hunting seasons included parts of Blue Ridge and Coopers Creek Wildlife Management Areas (WMAs, (**a**)) and private land (white areas in (**a**), [20]). WMAs are located in the northern region of Georgia, USA (**b**), within the Chattahoochee National Forest ((**c**); [21,22]).

We captured deer and analyzed their movements on a 135 km^2^ study area that included private land (11%) and public land (89%) in northern Georgia, USA (Figure 1).

The public land portion consisted of Chattahoochee National Forest land, 76% of which was Blue Ridge and Coopers Creek Wildlife Management Areas (WMAs). WMAs were managed cooperatively; the Georgia Department of Natural Resources maintained wildlife openings (i.e., food plots), set hunting regulations, and conducted hunts, while the U.S. Department of Agriculture Forest Service conducted the remaining management activities, including timber harvest and prescribed fire [23]. 

The study area, which was located within the Blue Ridge physiographic province of the Appalachian Mountain Range, had elevations ranging from 570 to 1101 m ($\overset{-}{x}$ = 760, SD = 95) and slopes ranging from 0 to 56 degrees ($\overset{-}{x}$ = 17, SD = 8; [24]). The land cover of the study area was 95% forested (44% mixed forest, 39% deciduous forest, and 12% evergreen forest) and 5% open lands [25], which included 35 wildlife openings [26]. Wildlife openings were located across both WMAs to provide food sources for wildlife. Cool season openings were managed as perennial plantings to provide winter nutrition and act as buffers during poor mast years, while warm season openings were planted to annual crops to provide summer nutrition. Twenty-four percent of the study area contained mountain laurel (*Kalmia latifolia*) or rhododendron (*Rhododendron maximum*) understories [27]. 

Deer population declines in northern Georgia WMAs became evident during the early 2000s [18,19]. Simultaneously, populations of black bears (*Ursus americanus*; [28]), coyotes (*Canis latrans*; [29,30]), bobcats (*Lynx rufus*; [31]), and wild pigs (*Sus scrofa*; [32]) increased, and forests matured [18]. Deer population declines in the study area were attributed to an extremely low fawn survival rate (16% survival of 71 fawns monitored to 12 weeks of age during 2018–2020; [33]), primarily due to predation by black bears, coyotes, and bobcats [34]. The lack of early successional plant communities and early forest stages [18] that provide essential food and cover for deer was linked to fawn predation, low fawn survival, and resultant deer population decline [33,34]. 

Each WMA hosted one primitive weapons hunt (4–5 days), two modern firearms hunts (4–7 days each), and 4–5 weeks of archery hunting during the 2018–2019 and 2019–2020 seasons. During the 2018–2019 season, most WMA hunts were antlered deer-only with a few limited opportunities for antlerless harvest, whereas during the 2019–2020 season, WMA hunts were antlered deer-only. The harvest of black bears and wild pigs was permitted during all deer hunts. There was no limit to the number of hunters who could hunt on the WMAs. On private land, either-sex archery deer hunting occurred from mid-September to mid-January. Private land muzzleloader and firearms hunting occurred from mid-October to mid-January with alternating weeks of either-sex and antlered deer-only opportunities. Legal hunting hours were 30 min before sunrise until 30 min after sunset.

### 2.2. Deer Capture and Data Analysis

We captured 26 female white-tailed deer (≥1.5 years) during 1 January–31 March in 2018 and 2019 using drop nets, rocket nets, Clover traps, and dart projectors on sites baited with whole kernel corn. We fitted each female deer with a GPS radio collar (VERTEX Plus Iridium V 3.2, Vectronic Aerospace GmbH, Berlin, Germany) that recorded locations every 30 min during our study period. Our study period included pre-hunt, hunt, and post-hunt periods relative to 7 public land firearms hunts that occurred on Blue Ridge and Coopers Creek WMAs during the 2018–2019 and 2019–2020 hunting seasons. 

We chose firearms hunts for our analysis because the most hunting pressure occurred during those hunts relative to primitive weapons and archery. We excluded one firearms hunt on Coopers Creek WMA due to a lack of deer GPS data. Firearms hunts typically lasted 4 days, with a range of 4–7 days. Each pre-hunt and post-hunt period equaled the length of the corresponding hunt and occurred immediately before and after the hunt, respectively. 

Because we only investigated deer responses to WMA hunting, we excluded deer with home ranges entirely on private land during the study period (*n* = 6) from our analyses. We filtered all location datasets to remove extreme outliers with >10.0 GPS dilution of precision [12,35]. We used the dynamic Brownian bridge movement model [36] by implementing the brownian.bridge.dyn function in the move package in program R [37]. This model calculates an animal’s utilization distribution (UD) using estimated movement paths rather than assuming each location is independent of others [36]. UDs outline the space use of an animal by calculating the probability density of the animal’s frequency of occurrence. Each deer-hunt-period combination included roughly 190 locations as inputs for the model. The model parameters included a window size of nine locations, a margin of three locations, a resolution of 30 m, and a collar error of 20.8 m. We calculated the horizontal positional error of the GPS collars by comparing 67 total locations recorded by 2 collars while placed on top of a National Geodetic Survey benchmark in Blairsville, Georgia, USA, to the actual benchmark coordinates. We used the distance in x and y from each recorded location to the true location to determine a 95% confidence interval root mean square error [38].

We analyzed differences in nine variables involving deer movement and space use among pre-hunt, hunt, and post-hunt periods:

- Diurnal movement and nocturnal movement: We calculated movements during shooting hours (diurnal) and non-shooting hours (nocturnal) by averaging step length, which was the distance in meters between successive 30 min locations. Prior to averaging, we removed step length values from the dataset that corresponded with time intervals outside of 25–35 min, which accounted for <5% of the total step length values.
- 50% UD and 90% UD: To characterize space use, we calculated the size of 50% UDs to represent the core area of use and 90% UDs to represent the broader area of use.
- Hunter Selection: We used ArcGIS Pro 2.8 (Environmental Systems Research Institute, Inc., Redlands, CA, USA) to calculate the average relative probability of hunter selection within 90% UDs. This is based on Rosenberger et al. [39], where the relative probability of a hunter selecting each 30 m pixel across the WMAs was estimated based on elevation, slope, three forest cover types, and distance to roads, trails, and openings. On the WMA scale, hunters showed a stronger preference for lower elevations and areas closer to deciduous forests relative to the other covariates.
- Openings: We used ArcMap 10.7.1 (Environmental Systems Research Institute, Inc., Redlands, CA, USA) to calculate the average Euclidean distance of the 90% UD to a wildlife opening [26].
- Public Land: We used ArcMap 10.7.1 to calculate the proportion of each 90% UD that contained public land [22].
- Deciduous: We used ArcMap 10.7.1 to calculate the proportion of each 90% UD that contained deciduous forest [25].
- Understory: We used ArcMap 10.7.1 to calculate the proportion of each 90% UD that contained a rhododendron or mountain laurel understory [27].

We tested the effect of the period (i.e., pre-hunt, hunt, and post-hunt periods) on each set of variables using mixed effects models via the lme function in the nlme package [40] in program R [37]. The period was the fixed effect, and the collar ID, month, and year were random effects. Collar ID accounted for variation among individual deer, month accounted for variation with the progression of time within a hunting season (each period was classified by the corresponding month of its start date), and year accounted for variation between the hunting seasons (i.e., 2018–2019 or 2019–2020). Before running the models, we transformed proportion and probability variables by taking the arcsine of the square root of the value. We performed an ANOVA on model output and Tukey’s HSD Multiple Comparisons test on variables with significant *p*-values from the ANOVA using the multcomp package [41] in program R [37]. We used an alpha level of 0.05 to determine statistical significance and back-transformed variables to their original units for interpretation of results.

## 3. Results

We analyzed the movements of 20 adult female deer that provided data for 44 deer-hunt combinations (Table 1). 

We did not detect differences in step length, nor did we detect differences in size or composition of 90% UDs, among pre-hunt, hunt, and post-hunt periods (Table 2). We detected a statistically significant difference in the size of 50% UDs between pre-hunt and hunt periods (*Z* = 2.436, *p* = 0.039) and between pre-hunt and post-hunt periods (*Z* = 2.385, *p* = 0.045). Pre-hunt 50% UDs ($\overset{-}{x}$ = 7.0 ha, SE = 0.4 ha) were slightly larger than both hunt ($\overset{-}{x}$ = 6.0 ha, SE = 0.3 ha) and post-hunt ($\overset{-}{x}\;$= 6.0 ha, SE = 0.2 ha) 50% UDs.

Collar ID explained the most random variation in the 90% UD composition-related variables, including public land, understory, hunter selection, deciduous, and distance to openings. Month explained the most random variation in diurnal and nocturnal movement, as well as size of 50% and 90% UDs (Table 3). 

## 4. Discussion

Our results indicated that firearms hunts did not cause significant changes in the movements of female deer. Eight of the nine movement and space use-related variables we evaluated did not differ among pre-hunt, hunt, and post-hunt periods. Deer movement rates are more likely to fluctuate in response to high hunter densities [11], and hunter density was consistently low in our study area. Over our study period, the most attended hunt occurred on Blue Ridge WMA (9285 ha), where 266 hunters checked in, representing 1 hunter for every 35 ha of land available for hunting. Based on the land area to hunter ratio, there was only 1 hunter for every deer’s 90% UD. Furthermore, Rosenberger et al. [39] found that hunting pressure was not uniform across the same study area. While hunters on the WMAs in our study area selected areas closer to deciduous forest and wildlife openings [39], the 90% UDs of female deer only averaged 17% deciduous forest and were an average distance of 553 m from a wildlife opening. Overall, the average relative probability of hunter selection within the 90% UDs was 27%. As a result, the deer in our study likely had even less exposure to hunters than the hunter-to-land area ratio suggests. Therefore, deer likely did not utilize different areas or change their movements because they were already utilizing areas with little to no hunting pressure. De facto refugia are areas open to hunting that serve as refuges for game because of their physical characteristics (e.g., slope; [43]). Deer in our study area likely had ample de facto refugia within their 90% UDs due to the rugged terrain. Furthermore, on average, 27% of each deer’s 90% UD contained rhododendron or mountain laurel. These shrub species represented the only consistent source of cover for deer during the hunting season within the homogenous, closed-canopy landscape of our study area. The dense understory cover provided by rhododendron and mountain laurel may have aided the ability of deer to hide from the occasional hunter while remaining within their 90% UD area [3]. Similarly, Lone et al. [9] found that female red deer did not shift their habitat use in response to hunting because they were already largely using areas of dense cover before hunting started. 

Female deer movement did not differ among pre-hunt, hunt, and post-hunt periods in our study, even when accounting for shooting hours versus non-shooting hours. Other studies with greater levels of hunting pressure have documented deer responding to hunters on the landscape by decreasing their diurnal movements and increasing their nocturnal activity [5,11,44,45]. The usage of public land (i.e., WMAs) versus privately owned land by deer in our study also did not differ among the periods, contrary to a few other studies with greater hunter densities that found elk shifted onto private land refuge areas at the onset of public land hunts [46,47]. The duration of hunts is also an important consideration. Kilpatrick and Lima [48] suggested that deer behavioral responses to hunting pressure may decrease if a series of short, intense hunt periods are separated by periods of no hunting. Firearms hunts in our study area were infrequent (two hunts per WMA per season) and short in duration (4–7 days each).

The size of 50% UDs was the only variable that statistically differed among periods. On average, pre-hunt 50% UDs were 1 ha larger than those during the hunt and post-hunt. Little et al. [11] and Marantz et al. [12] found that deer used smaller areas more intensively in response to hunting while also documenting decreases in overall deer movements. Potential reasons included concentrating and reducing movements to the most familiar areas within their range to increase the probability of avoiding risk. However, the deer in our study did not decrease their movements. And given that the size of 50% UDs was the only variable that statistically differed among periods and the magnitude of the difference was relatively small (1.0 ha), this result was likely not biologically significant. 

Although many studies have documented ungulate movement responses to hunting, a few studies reported similar results in which hunting pressure was insufficient to cause significant changes in ungulate movements [49,50,51]. A study in Maryland, USA, found that adult male white-tailed deer home range and core area size remained constant between pre-hunt and hunt periods, and decreases in overall movement and activity were attributed to the coincident transition from rut to post-rut rather than hunting activities [51]. A study of moose (*Alces alces*) in Sweden also concluded that alterations in female movements were due to rutting activity rather than hunting disturbance and that female moose perceived the risk of humans no differently than the constant risk of other predators [49]. Further, female white-tailed deer in Louisiana, USA, did not change their home ranges, core areas, or movement rates in response to small game hunting activities [50]. 

Where population sustainability is a concern for a species, it is important for managers to consider the potential implications of hunting pressure on non-target animals. Perceived risk and stress associated with human-related disturbance or predation have the potential to negatively impact foraging and reproduction through avoidance of key habitats [9], increased vigilance [52,53], altered breeding-related movements from the norm [11], or disruption of ovulation [14]. These factors can ultimately negatively affect body condition, survival, and fecundity [6,7,54], which are all key to a struggling population. Therefore, indirect impacts of hunting should be considered with the same importance as direct impacts of hunting (i.e., mortality) for population sustainability. Our study only investigated movement-related responses of female deer, but it is important to consider that indirect effects of hunting pressure may occur outside the realm of deer location data, such as increased levels of stress hormones detected in ungulates from predation risk [54] and other human-related disturbances [55,56].

## 5. Conclusions

Overall, our results suggest that the low level of hunting pressure in our study area and lack of exposure to hunters led to no biologically significant changes in female deer movements during our study period. The deer population in the mountains of northern Georgia, USA, has reached a critically low level, and, starting in 2019, only antlered deer (i.e., males) were legally available for harvest. To the extent of the findings presented in this paper, adjustments to the management of hunting in our study area do not appear to be necessary for the purpose of minimizing hunting-related disturbance on female deer. However, managers should continue to consider female deer behavior when evaluating future changes to hunting regulations.

## Figures and Tables

**Table 1:** Movements of 20 GPS-collared female white-tailed deer were compared among pre-hunt, hunt, and post-hunt periods relative to 7 firearms hunts on Blue Ridge (BR) and Coopers Creek (CC) Wildlife Management Areas (WMAs) in northern Georgia, USA, during the 2018–2019 and 2019–2020 hunting seasons. Hunts were open to antlered deer only unless otherwise noted. All seven hunts took place in the same study area; thus, data from GPS-collared deer were analyzed collectively.

Season	WMA	Pre-Hunt Dates	Hunt Dates	Post-Hunt Dates	Number of Hunters ^1^	Number of Hunters per 100 ha ^2^	Number of GPS-Collared Deer
2018–2019	BR	20–23 Oct	24–27 Oct	25–28 Oct	95	1.0	7
2018–2019	BR	20–23 Nov	21–24 Nov ^3^	25–28 Nov	266	2.9	7
2018–2019	CC	24–27 Nov	28 Nov–01 Dec	02–05 Dec	226	1.9	1
2019–2020	BR	19–22 Oct	23–26 Oct	27–30 Oct	78	0.8	9
2019–2020	BR	23–26 Nov	27–30 Nov	01–04 Dec	186	2.0	16
2019–2020	CC	22–26 Nov	27 Nov–01 Dec	02–06 Dec	159	1.3	2
2019–2020	CC	19–25 Dec	26 Dec–01 Jan	02–08 Jan	228	1.9	2

^1^ Number of hunters who checked in or signed in to hunt. ^2^ Calculated using the total number of hunters who checked in/signed in and the total land area of the WMA. Actual hunter density likely differed depending on hunter distribution. ^3^ Last day was open to either sex deer.

**Table 2:** Nine movement and space use variables for 20 GPS-collared female white-tailed deer were compared among pre-hunt, hunt, and post-hunt periods relative to 7 firearms hunts on Blue Ridge and Coopers Creek Wildlife Management Areas in northern Georgia, USA, during the 2018–2019 and 2019–2020 hunting seasons. Movement (i.e., mean step length), 90% utilization distributions (UDs), and 50% UDs were calculated for each period with 30 min GPS locations using the dynamic Brownian bridge movement model for 44 deer-hunt combinations.

Variable Name	Variable Explained	Biological Meaning	$\mathrm{Pre}\text{-}\mathrm{Hunt}\;\overset{-}{x}$ (SE)	$\mathrm{Hunt}\;\overset{-}{x}$ (SE)	$\mathrm{Post}\text{-}\mathrm{Hunt}\;\overset{-}{x}$	*F*	*p*
90% UD	Area (ha) of 90% utilization distribution ^1^	Size of short-term home range	36 (2)	32 (1)	34 (3)	2.73	0.07
Public Land	Proportion of 90% UD containing public land (i.e., WMA) ^2^	Space use of the WMA	0.93 (0.03)	0.92 (0.03)	0.92 (0.03)	0.64	0.53
Understory	Proportion of 90% UD containing rhododendron or mountain laurel understory ^3^	Cover	0.27 (0.02)	0.27 (0.02)	0.27 (0.02)	0.06	0.94
Hunter Selection	Average relative probability of hunter selection within 90% UD ^4^	Spatial preference by hunters	0.28 (0.02)	0.26 (0.02)	0.28 (0.03)	0.53	0.59
Deciduous	Proportion of 90% UD containing NLCD 2016 land cover class deciduous forest ^5^	Food/hunter preference	0.16 (0.02)	0.17 (0.03)	0.17 (0.02)	0.23	0.80
Openings	Average Euclidean distance (m) of 90% UD to wildlife openings ^6^	Food/hunter preference	538 (46)	550 (41)	571 (46)	1.04	0.36
50% UD	Area (ha) of 50% utilization distribution	Size of short-term core area	7.0 (0.4)	6.0 (0.3)	6.0 (0.2)	3.84	0.03
Diurnal Movement	Mean step length (m) during legal shooting hours	Temporal movement response to hunters	87 (6)	78 (3)	80 (4)	1.78	0.18
Nocturnal Movement	Mean step length (m) during non-shooting hours	Temporal movement response to lack of hunters	75 (7)	66 (2)	68 (4)	1.18	0.32

^1^ A utilization distribution outlines the space use of an animal by calculating the probability density of their frequency of occurrence [36]. ^2^ U.S. Department of Agriculture Forest Service [42]. ^3^ J. Hepinstall-Cymerman [27]. ^4^ Rosenberger et al. [39]. ^5^ U.S. Geological Survey [25]. ^6^ E. Mavity [26].

**Table 3:** Nine movement and space use variables for 20 GPS-collared female white-tailed deer were compared among pre-hunt, hunt, and post-hunt periods relative to 7 firearms hunts on Blue Ridge and Coopers Creek Wildlife Management Areas in northern Georgia, USA, during the 2018–2019 and 2019–2020 hunting seasons. Collar ID, Year, and Month were incorporated as random effects into linear mixed effects models. Standard deviations for each random effect are shown below by variable.

Variable (Units)	Collar ID	Year	Month
90% UD (ha)	<0.01	<0.01	12
Public Land (%)	0.13	<0.01	<0.01
Understory (%)	0.02	<0.01	<0.01
Hunter Selection (%)	0.05	<0.01	<0.01
Deciduous (%)	0.04	<0.01	<0.01
Openings (m)	271	<0.01	48
50% UD (ha)	<0.01	<0.01	1
Diurnal Movement (m)	<0.01	9	16
Nocturnal Movement (m)	5	<0.01	13

## Data Availability

The data presented in this study are available on request from the corresponding author. The data are not publicly available in an effort to not expose individual locations of study animals that are part of a limited population.
